# Editorial: Insights in renal endocrinology: 2021

**DOI:** 10.3389/fendo.2022.1003683

**Published:** 2022-09-20

**Authors:** Nehal M. Elsherbiny, Eman Said

**Affiliations:** ^1^ Department of Biochemistry, Faculty of Pharmacy, Mansoura University, Mansoura, Egypt; ^2^ Department of Pharmaceutical Chemistry, Faculty of Pharmacy, University of Tabuk, Tabuk, Saudi Arabia; ^3^ Department of Pharmacology and Toxicology, Faculty of Pharmacy, Mansoura University, Mansoura, Egypt; ^4^ Faculty of Pharmacy, New Mansoura University, New Mansoura, Egypt

**Keywords:** renal endocrine, biomolecules, renal function, prognosis, diagnosis

Renal endocrinology includes biochemical, pharmacological, physiological, and pathophysiological aspects of hormones and autacoids released by the kidney, in addition to nonrenal hormones that have effects on renal function (1). Various hormones are produced/activated by the kidney such as erythropoietin, renin-angiotensin system, and calcitriol. Moreover, the kidney produces local hormones such as prostaglandins and adrenomedullin, and enzymes such as kallikreins, which cleave pro-hormones in other distant sites. Additionally, the kidney is a primary target for many hormones like the natriuretic peptides, angiotensin, and aldosterone. Disturbance in the renal endocrine system is encountered in primary acute and chronic kidney diseases and observed in many other diseases such as diabetes mellitus. Of note, other hormonal abnormalities such as thyroid, parathyroid disorders, and acromegaly can affect renal function and structure (2). The present Research Topic aims to provide a platform for novel research in the field of renal endocrinology. The Research Topic includes four original research articles which provide insights into relationships between endogenous biological factors and indicators of renal function, that may possess pathogenic roles in various renal endocrinology disorders. Understanding these relationships would help in the development of better diagnostic, prognostic and/or therapeutic strategies for various diseases associated with renal endocrinology diseases.

Hypertension is a known risk factor for white matter lesions (WMLs). WMLs are vascular pathologies that affect the small cerebral blood vessels because of hypoperfusion, ischemia, blood-brain barrier breakdown, endothelial dysfunction, inflammation, or genetic factors. These lesions increase the risk of cognitive decline, depression, stroke incidence and recurrence, and even death (3). Indeed, hypertensive patients are highly susceptible to the development of cerebral diseases, and inhibition of renin-angiotensin system (RAS) has been reported to attenuate cognitive deficits associated with aging, Parkinson’s disease, and Alzheimer’s disease (4). However, about 30% of hypertensive patients display a low-renin profile (5). Further, increased aldosterone secretion, as well as activation of the mineralocorticoid receptor, have been reported in patients with low-renin hypertension (6) and many preclinical studies demonstrated an association between aldosterone and WMLs (7, 8). The first article in the present Research Topic Yuan et al. aimed to analyze the association between plasma aldosterone level, plasma renin activity, and WMLs in patients with hypertension. The authors demonstrated that higher plasma aldosterone and lower plasma renin activity increased the risk of WMLs.

Diabetic nephropathy (DN) is a known and serious microvascular complication of diabetes mellitus, a rising global public health problem (9). Up to 40% of patients with Type 2 DM develop (DN) (10). Currently, albuminuria and/or declined estimated glomerular filtration rate (eGFR) represent the commonly used diagnostic criteria for DN (11). The eGFR equations have been found to be less accurate in diabetic patients compared to non-diabetic individuals. However, hemoglobin A1c (HbA1c) was an independent factor associated with eGFR equations accuracy (12). HbA1c has been widely used in clinical practice to assess average blood glucose levels two to three months prior to biochemical analysis. Hence, it is a good indicator of glycemic control (13). The second article in the present Research Topic (An et al.) studied the relation between eGFR changes and HbA1c in Type 2 DM patients. The trend of eGFR change was decreased alongside HbA1c reduction and this was independent of the duration of diabetes mellitus, urinary albumin-to-creatinine ratio, and hyperfiltration. The authors recommended sustained monitoring and cautious interpretation of HbA1c and eGFR changes for Type 2 DM patients.

Homocysteine is an intermediate product of the methionine cycle and cysteine metabolism (14). Several studies have linked HHcy, elevated blood levels of homocysteine, to the incidence and progression of cardiovascular and cerebrovascular diseases. Indeed, Hcy has been reported to induce vascular endothelial damage (15, 16). Estrogen is a fat-soluble steroidal sex hormone. E2 is the most active form of estrogen and is the form responsible for maintaining sexual and reproductive functions, regulating the maturation of female organs, controlling lipid metabolism, and protecting the cardiovascular system (17). E2 has been suggested to regulate homocysteine metabolism and postmenopausal women possess low estrogen but elevated homocysteine (18). In renal diseases, estrogen administration has demonstrated renal protective efficacies (19). Of note, many enzymes that regulate homocysteine metabolism exist in the kidney (20). Therefore, the third article in the current Research Topic Niu et al. studied the relationship between E2, renal biomarkers, and homocysteine in female patients with HHcy. The authors observed a positive relationship between serum creatinine, urinary albumin, and homocysteine, but a negative relationship between E2, GFR, and homocysteine, suggesting a protective effect of E2 against HHcy. Also, the mediating effect models demonstrated the implication of urinary albumin and GFR in the relationship between E2 and homocysteine.

Dysregulated lipid metabolism characterized by increased triglycerides (TGs) and decreased high-density lipoprotein-C (HDL-C) has been linked to impaired renal function in chronic kidney diseases (21). TGs levels fluctuate based on nutritional status (22), and the sole use of HDL-C as a diagnostic biomarker is controversial (23). Therefore, the use of TG/HDL-C ratio has been found to be more reliable. The fourth article in the current Research Topic Pei et al. aimed to study the TG/HDL-C ratio as a predictor of Immunoglobulin A nephropathy (IgAN) progression. IgAN is the most common cause of primary glomerulonephritis and a leading cause of end-stage renal disease. Diffusely deposited Immunoglobulin A in the kidneys is a hallmark of this disease (24). The article demonstrated a strong association between a high TG/HDL ratio and worse renal survival in IgAN patients, highlighting the importance of using this ratio as a predictive marker for IgAN progression.

Overall, the articles outlined in the current Research Topic introduce new relationships between biological factors and indicators of renal function as pathogenic mediators, diagnostic, and/or prognostic approaches for better management of common renal endocrinology disorders.

## Author contributions

Conceptualization: NE; Writing: NE and ES; Review and Editing: NE and ES. All authors contributed to the article and approved the submitted version.

## Conflict of interest

The authors declare that the research was conducted in the absence of any commercial or financial relationships that could be construed as a potential conflict of interest.

## Publisher’s note

All claims expressed in this article are solely those of the authors and do not necessarily represent those of their affiliated organizations, or those of the publisher, the editors and the reviewers. Any product that may be evaluated in this article, or claim that may be made by its manufacturer, is not guaranteed or endorsed by the publisher.
